# Anesthetic Management of an Elective Cesarean Section in a Patient With Systemic Mastocytosis and Latex Allergy, Complicated by Incidental Intraoperative Wolff-Parkinson-White Pre-excitation: A Case Report

**DOI:** 10.7759/cureus.112209

**Published:** 2026-07-07

**Authors:** Giovanni Maggio, Daniele S Paternò, Marianna Tiralongo, Giuseppe Scibilia, Massimiliano Sorbello

**Affiliations:** 1 Department of Anesthesia and Intensive Care, Giovanni Paolo II Hospital, Ragusa, ITA; 2 Department of Obstetric and Gynecological Surgery, Giovanni Paolo II Hospital, Ragusa, ITA

**Keywords:** cesarean section, latex allergy, mast cell activation, perioperative management, spinal anesthesia, systemic mastocytosis, tryptase, wolff-parkinson-white syndrome

## Abstract

Systemic mastocytosis (SM) is a rare clonal mast cell disorder in which physical, pharmacological, or emotional triggers can provoke massive mediator release and life-threatening anaphylactoid reactions. Pregnancy and delivery are recognized as high-risk periods that demand careful multidisciplinary planning. Cardiovascular manifestations of mast cell activation disease are increasingly recognized; however, their interplay with primary conduction abnormalities is poorly characterized, and the available evidence is largely limited to isolated case reports. We describe the elective cesarean delivery of a 40-year-old woman with indolent SM and documented latex allergy, managed within a multidisciplinary framework. After corticosteroid and antihistamine premedication in a latex-free operating room, spinal anesthesia was performed without intrathecal opioids; spinal-induced hypotension was treated with phenylephrine boluses, and postoperative analgesia relied on paracetamol alone, with nonsteroidal anti-inflammatory drugs (NSAIDs) deliberately avoided. Serial serum tryptase concentrations remained within the normal range throughout the perioperative period (baseline 7.9 µg/L; intraoperative 10.1 µg/L; early postoperative 9.3 µg/L), with no value reaching the consensus threshold for significant mast cell activation, supporting the effectiveness and safety of the adopted strategy. Unexpectedly, a new Wolff-Parkinson-White (WPW) pre-excitation pattern appeared intraoperatively in a patient with a normal antepartum tracing. She remained asymptomatic, and the subsequent cardiac workup was unremarkable, with outpatient electrophysiological follow-up arranged. This case highlights the value of a structured, prevention-oriented anesthetic strategy in SM, supports spinal anesthesia with hyperbaric bupivacaine as a reasonable preferred option when not contraindicated, and broadens the spectrum of perioperative electrocardiographic findings that may be observed in this population.

## Introduction

Systemic mastocytosis (SM) is a clonal mast cell disorder characterized by the pathological accumulation of neoplastic mast cells bearing the KIT D816V mutation, most commonly in the bone marrow. It ranges from indolent SM - the most prevalent form, with a near-normal life expectancy - to more aggressive variants [1,2]. Its central pathophysiological feature, regardless of subtype, is the propensity of neoplastic mast cells to degranulate in response to a wide range of triggers, releasing vasoactive and pro-inflammatory mediators (histamine, tryptase, prostaglandin D2, leukotrienes) that can precipitate reactions ranging from anaphylactoid events to cardiovascular collapse [3,4].

This reactivity is of particular concern to the anesthesiologist because many perioperative agents - including opioids, neuromuscular blocking agents, NSAIDs, and certain hypnotics - are recognized mast cell activators, while mechanical and sympathoadrenal stimuli such as laryngoscopy further compound the risk [2,3,5]. The challenge is amplified in the peripartum setting, where the risk of severe mast cell-related reactions is significantly increased during labor and delivery, and where available guidance is confined to isolated case reports and expert opinion, with no prospective data [6-8]. Each new report, therefore, represents a valuable contribution to the literature [5].

Against this background, we report the perioperative management of a patient with indolent SM and latex allergy undergoing elective cesarean section. The novelty of this report is twofold: First, it documents an integrated, prevention-oriented anesthetic strategy whose effectiveness is objectively supported by serial serum tryptase measurements. Second, it describes the unexpected intraoperative appearance of a new Wolff-Parkinson-White (WPW) pre-excitation pattern in a patient with a normal antepartum electrocardiogram (ECG) - an incidental finding not previously reported in this setting.

## Case presentation

Clinical history and preoperative assessment

A 40-year-old woman (gravida 3, para 0; weight: 60 kg, height: 165 cm, body mass index: 22.0 kg/m²) presented at 38 weeks and 0 days of gestation for elective cesarean section. The patient had received a diagnosis of SM several years earlier, confirmed by bone marrow biopsy demonstrating multifocal dense aggregates of atypical tryptase-positive mast cells carrying the KIT D816V mutation. Her disease had been classified as indolent SM, and she was under active hematological follow-up on a long-term antimediator regimen [1,2].

Her allergological history was extensive and clinically significant: she had documented systemic reactions - including urticaria, angioedema, and on one occasion bronchospasm - to multiple antibiotic classes, nonsteroidal anti-inflammatory drugs (NSAIDs), and several foods. Latex allergy was confirmed by specific IgE testing and a consistent clinical history, with reactions reported after exposure to latex gloves and medical devices. No previous anesthetic records were available, as the current pregnancy represented her first surgical exposure. A preoperative 12-lead ECG obtained during the antepartum assessment demonstrated normal sinus rhythm without conduction abnormalities, a normal PR interval, and no evidence of pre-excitation. Routine antepartum laboratory investigations, including serum electrolytes, renal function, and a baseline serum tryptase, were within normal limits.

The cesarean section was indicated for obstetric reasons, and the decision to proceed electively rather than await spontaneous labor was made jointly by the obstetric and anesthesiology teams in light of the patient's risk profile, which favored a controlled and fully prepared setting over an emergency procedure [6,8]. The multidisciplinary perioperative plan was developed in close collaboration among the departments of obstetrics, anesthesiology, hematology, and neonatology, with additional input from the referring hematological center of excellence [3,6]. A formal pre-anesthetic assessment was conducted several days before the planned procedure, during which all potential pharmacological triggers were systematically reviewed, and an individualized perioperative protocol was defined. This approach mirrors the recommendations of published expert consensus documents for the management of patients with SM undergoing surgical procedures [3,9].

Preoperative preparation and premedication

The premedication protocol, formulated in agreement with the hematology team, was designed to pharmacologically stabilize mast cells before the anticipated procedural stimuli [3,9]. On the morning of surgery, the patient received prednisone 60 mg orally to attenuate the inflammatory and immunological amplification cascade that could follow mast cell activation. Chlorphenamine, an H1 antihistamine, was administered to block histamine receptor-mediated effects at the tissue level and was planned to be continued throughout the postoperative period. This combined corticosteroid and antihistamine premedication regimen is consistent with the recommendations of the Spanish Network on Mastocytosis (REMA), which has demonstrated its safety and efficacy in reducing perioperative mast cell activation in adult and pediatric patients with SM undergoing invasive procedures [9].

The operating room was prepared with meticulous attention to the elimination of all latex-containing materials. All gloves, intravenous tubing, syringes, airway adjuncts, and any other potentially latex-containing equipment were replaced with certified latex-free alternatives [3]. All members of the operative team were explicitly briefed on the latex-free requirement before entering the theater. Emergency drugs - including intramuscular adrenaline 1:1000, intravenous hydrocortisone, and additional antihistamines - were drawn up, labeled, and placed within immediate reach at the anesthetic workstation before the patient entered the room [3].

Anesthetic technique

The selection of the anesthetic technique was among the most consequential decisions in the perioperative planning of this case. General anesthesia was deliberately excluded for several converging reasons. Laryngoscopy and tracheal intubation are potent mechanical stimuli with a well-documented potential to trigger mast cell degranulation through both direct physical and sympathoadrenal mechanisms [5,9]. Neuromuscular blocking agents are among the pharmacological drug classes most frequently implicated in perioperative anaphylaxis and are recognized mast cell activators [10]. Volatile halogenated agents have been associated with histamine release and hemodynamic instability in sensitive individuals, and severe anaphylaxis in patients with SM has been reported even with newer-generation intravenous hypnotics such as remimazolam [5,11]. The risk of uncontrolled mast cell activation under general anesthesia, in a patient with already heightened systemic reactivity, was deemed unacceptable when a safe locoregional alternative was available [5].

Spinal anesthesia was therefore chosen as the technique of election, consistent with the predominant recommendations in the literature for this patient population [5,8,12]. Retrospective evidence on the safety of local anesthetics in patients with clonal mast cell disease supports a low rate of hypersensitivity reactions when amide-type agents are used [13]. The subarachnoid block was performed with the patient in the sitting position using a 25-gauge Whitacre pencil-point needle at the L3-L4 interspace. Hyperbaric bupivacaine (0.5%) was administered at a dose of 12 mg (2.4 mL), without the addition of intrathecal opioids - a deliberate choice, as morphine is known to cause direct, non-immunological histamine release from mast cells [14,15], and the benefit-risk balance in this context did not favor its use. Amide-type local anesthetics, and hyperbaric bupivacaine in particular, are considered the agents of choice for neuraxial anesthesia in patients with SM owing to their low histamine-releasing potential; moreover, the baricity and dose employed are well within the established range for elective cesarean section [13,14]. The block was established rapidly and uneventfully, achieving an adequate sensory level for the planned procedure. Maintenance of consciousness throughout the procedure was an additional advantage of the locoregional approach, enabling real-time clinical monitoring for early signs of systemic mast cell activation.

Intraoperative management

Intraoperative monitoring consisted of continuous three-lead electrocardiography, pulse oximetry, and non-invasive arterial blood pressure measurement at two-minute intervals. As is routinely encountered with spinal anesthesia for cesarean section, sympathetic blockade resulted in a clinically significant episode of hypotension, which was managed effectively with phenylephrine administered as intravenous boluses titrated to hemodynamic response.

The choice of phenylephrine as the vasopressor in this patient was both evidence-based and pathophysiologically reasoned [5]. As a selective alpha-1 adrenergic agonist devoid of beta-adrenergic activity, phenylephrine restores vascular tone through pure vasoconstriction without inducing tachycardia or indirect sympathomimetic stimulation. This profile is particularly advantageous in patients with SM, in whom the sympathoadrenal activation and reflex tachycardia associated with ephedrine could theoretically contribute to mast cell stimulation [5]. The bolus strategy allowed precise hemodynamic titration, and the response was satisfactory throughout.

Perioperative antibiotic prophylaxis was performed with cefazolin, a first-generation cephalosporin selected for its favorable prophylactic profile in cesarean section and its low histamine-releasing potential [5,9]. The patient's history of reactions to multiple antibiotic classes required careful consideration; cefazolin was chosen after allergological review confirmed the absence of documented cross-reactivity. For the prevention of uterine atony, oxytocin was administered intravenously, delivered slowly, and in adequate dilution to minimize its known vasoactive effects [5]. The neonatologist was present in the operating room from the outset, at the neonatal resuscitation station, in recognition of the potential neonatal implications of maternal SM and the agents employed perioperatively [6].

Serum tryptase - the primary enzymatic marker of mast cell degranulation and the reference biochemical standard for diagnosing anaphylaxis and assessing systemic mast cell activation [16,17] - was measured at multiple perioperative time points: a baseline value before initiation of the anesthetic (7.9 µg/L) and subsequent samples collected intraoperatively (10.1 µg/L) and in the early postoperative period (9.3 µg/L). All values remained within the normal reference range, providing objective biochemical evidence of the absence of clinically significant mast cell degranulation. According to the current consensus, a peak tryptase value exceeding 1.2 × baseline + 2 µg/L (equivalent to 11.5 µg/L in this patient; tryptase concentrations expressed in µg/L are numerically equivalent to ng/mL) is considered significant evidence of acute mast cell activation [17]. The absence of such elevation corroborates the clinical stability observed and supports the effectiveness and safety of the adopted strategy (Table 1).

**Table 1:** Perioperative medications and serial serum tryptase values. PO: per os (by mouth)

Time point	Intervention/measurement	Value
Preoperative (morning of surgery)	Prednisone PO/chlorphenamine (H1)	60 mg/standard dose
Baseline (antepartum)	Serum tryptase	7.9 µg/L
Spinal block	Hyperbaric bupivacaine (0.5%)	12 mg (2.4 mL)
Intraoperative (medications)	Phenylephrine boluses (titrated)/cefazolin/oxytocin	-
Intraoperative (tryptase)	Serum tryptase	10.1 µg/L
Early postoperative	Serum tryptase/paracetamol	9.3 µg/L/standard scheduled
Reference/threshold	Normal range/significant-activation cut-off	<11.4 µg/L/11.5 µg/L

**Figure 1:**
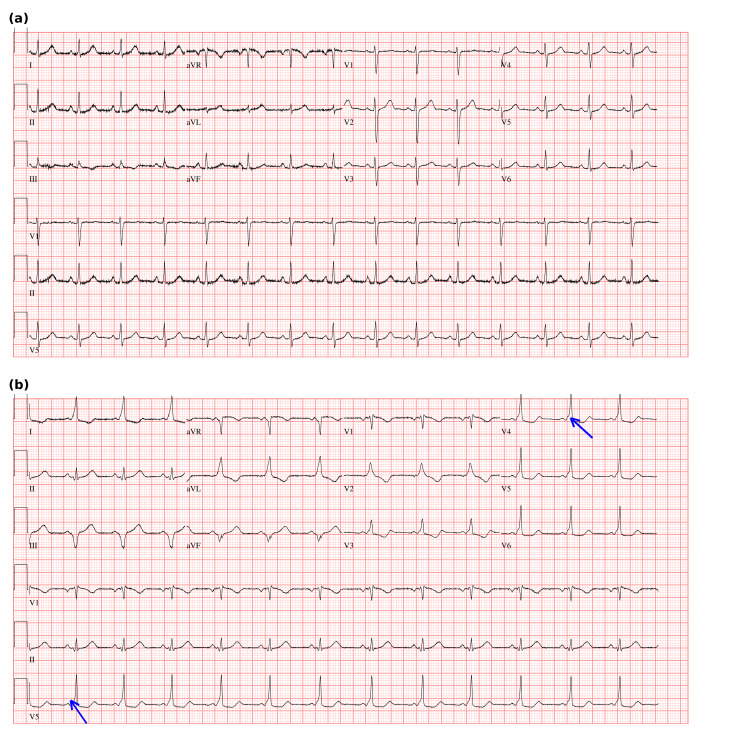
Twelve-lead electrocardiograms. (a) Antepartum tracing showing normal sinus rhythm, a normal PR interval, and no pre-excitation. (b) Intraoperative tracing recorded after wound closure, showing a short PR interval (<120 ms), a delta wave (indicated by arrows), and a widened QRS complex consistent with a Wolff-Parkinson-White pre-excitation pattern.

During the intraoperative period, a change in QRS morphology and an apparent shortening of the PR interval were noted on continuous rhythm-strip monitoring. A 12-lead ECG was therefore obtained intraoperatively and unexpectedly demonstrated a pre-excitation pattern consistent with the WPW syndrome, characterized by a short PR interval (<120 ms), a slurred upstroke of the QRS complex (delta wave), and secondary repolarization changes (Figure 1). Direct comparison with the antepartum tracing, which had shown sinus rhythm without pre-excitation, confirmed the finding to be new in onset. The patient remained completely asymptomatic, hemodynamically stable, and in sinus rhythm, with no tachyarrhythmia detected. In the absence of clinical or hemodynamic deterioration, surgery continued under close electrocardiographic surveillance, and the anesthetic plan was not modified.

The surgical procedure - a Pfannenstiel-type transverse laparotomy with lower-segment uterine incision - was completed without intraoperative complications. The intraoperative course was hemodynamically stable, with no clinical signs of mast cell activation at any point. A healthy newborn was delivered, with uneventful neonatal adaptation.

Postoperative management, cardiac workup, and analgesia

Following closure, the patient was transferred to the obstetric postoperative ward, where continuous monitoring of vital signs and telemetry was maintained for the initial postoperative hours. She remained hemodynamically stable and reported no symptoms suggestive of delayed mast cell activation, chest pain, palpitations, or dyspnea. The pre-excitation pattern detected intraoperatively persisted on serial postoperative ECGs without evolution into any tachyarrhythmia.

In light of the new finding, an urgent cardiology consultation was obtained. Serial high-sensitivity troponin assays were within normal limits, transthoracic echocardiography excluded structural cardiac disease and demonstrated preserved biventricular systolic function with no regional wall motion abnormalities, and serum electrolytes were unremarkable. In the absence of symptomatic tachyarrhythmias or evidence of acute ischemia, no antiarrhythmic therapy was initiated. The patient was counseled on the nature of the finding and on the warning symptoms that would require urgent reassessment, and was scheduled for outpatient electrophysiological follow-up, including risk stratification of accessory pathway conduction.

Postoperative analgesia required further pharmacological reasoning. NSAIDs, a cornerstone of multimodal analgesia in standard postoperative cesarean care, were categorically excluded: inhibition of cyclooxygenase by NSAIDs diverts arachidonic acid metabolism toward the 5-lipoxygenase pathway, enhancing leukotriene synthesis with consequent mast cell activation and a risk of bronchoconstriction and anaphylactoid reactions [3,5,9]. Postoperative pain was therefore managed exclusively with paracetamol at standard scheduled doses; paracetamol does not meaningfully inhibit peripheral cyclooxygenase under standard therapeutic conditions and is not associated with mast cell activation [5], and analgesia was judged clinically adequate. The antimediator regimen with chlorphenamine was continued postoperatively as planned [6]. The patient was discharged home in good clinical condition with planned outpatient follow-up by hematology, allergology, and cardiology.

## Discussion

This case illustrates the complexity and the potential for a successful outcome in the perioperative management of a patient with SM and concomitant latex allergy undergoing cesarean section. Several aspects warrant specific discussion.

Multidisciplinary team structure

The multidisciplinary team structure was arguably the single most important organizational determinant of outcome. The convergence of expertise from obstetrics, anesthesiology, hematology, allergology, and neonatology ensured that decisions were integrated across disciplines in a coherent, anticipatory framework [3,6,9]. Early involvement of the hematological center of reference allowed the anesthesiologist to benefit from disease-specific expertise unlikely to be available within routine institutional competency. This model of care, while resource-intensive, represents the standard consistently endorsed in the literature for patients with SM undergoing surgical procedures [3,6]. Published series confirm that the risk of severe intrapartum mast cell-related reactions is significantly increased during labor and delivery compared with the gestational period as a whole, and that adequate pre-delivery planning - including formal multidisciplinary consultation - is a key determinant of outcome [6,7].

Choice of anesthetic technique

The choice of spinal anesthesia over general anesthesia reflects the core principle of minimizing pharmacological and physical triggers in this population [5,8,12]. The literature, though limited to case series and expert consensus, is consistent in recommending neuraxial techniques as the preferred modality for patients with SM undergoing cesarean section [5,8,12], primarily because they circumvent tracheal intubation, neuromuscular blockade, and volatile agent exposure. The reported case of severe anaphylaxis induced by remimazolam in a mastocytosis patient further illustrates that even newer intravenous agents carry an unpredictable risk of mast cell activation [11], reinforcing the case for locoregional techniques whenever feasible. Hyperbaric bupivacaine (0.5%) at a dose of 12 mg provided effective surgical anesthesia while remaining within the established safety and efficacy range for elective cesarean delivery [14]. Retrospective evidence from a large series of patients with clonal mast cell disease confirms the favorable safety profile of amide-type local anesthetics, with a low prevalence of hypersensitivity reactions [13]. The deliberate omission of intrathecal opioids - in particular morphine - reflects its known capacity to induce histamine release through a non-IgE-mediated mechanism [15], a risk that outweighed the analgesic benefit in this context.

Hemodynamic management

The management of spinal hypotension with phenylephrine boluses deserves emphasis. Both phenylephrine and ephedrine are used for spinal hypotension during cesarean section, but their pharmacodynamic profiles differ in ways clinically relevant in SM. Ephedrine acts through indirect sympathomimetic mechanisms, producing a mixed alpha- and beta-adrenergic response that includes tachycardia; in a patient with SM, this sympathoadrenal activation carries a theoretical risk of mast cell stimulation. Phenylephrine, by contrast, produces vasoconstriction through direct and selective alpha-1 receptor agonism, without tachycardia or indirect catecholamine release, making it the pharmacologically preferable agent [5].

Biochemical validation: perioperative tryptase monitoring

The normality of perioperative serum tryptase values is a finding of considerable significance [16,17]. Tryptase is a serine protease stored almost exclusively in mast cell secretory granules and released into the systemic circulation during degranulation. Serial monitoring of tryptase in indolent SM correlates with disease activity and provides useful prognostic information [16]. In the perioperative setting, the consistent normality of tryptase values supports the effectiveness and safety of the preventive strategy in avoiding clinically significant mast cell activation. This biochemical endpoint strengthens the report beyond purely clinical observation and may serve as a model for outcome measurement in future cases [16,17].

Postoperative analgesia

The exclusion of NSAIDs from the postoperative regimen, with exclusive reliance on paracetamol, illustrates the need for a fundamentally reconsidered approach to pain management in patients with SM [3,5]. In standard obstetric practice, multimodal analgesia after cesarean section typically incorporates scheduled NSAIDs; in SM, however, the cyclooxygenase-inhibitory mechanism shared by all NSAIDs renders them pharmacologically incompatible with safe perioperative care [3,5]. The adequate analgesia achieved with paracetamol alone is reassuring and consistent with previous obstetric reports of similar patients [8,12].

Unexpected intraoperative pre-excitation: pathophysiological considerations

An unexpected and clinically intriguing element of this case was the appearance of a new electrocardiographic pre-excitation pattern intraoperatively, in the absence of any preoperative evidence of accessory pathway conduction. This was an entirely incidental finding, and the following mechanistic considerations are offered as hypotheses rather than as established causal explanations. WPW syndrome is conventionally regarded as a congenital conduction anomaly arising from an accessory atrioventricular pathway [18]; because the anatomical substrate is congenital, its de novo manifestation in an adult warrants consideration of alternative explanations. Three mechanistic hypotheses were considered.

The first, and most plausible hypothesis, is the unmasking of a pre-existing, intermittently conducting accessory pathway by perioperative autonomic and pharmacological influences. Conduction across an accessory pathway can be modulated by changes in sympathetic tone and sinus rate. The timing of the finding - emerging after spinal-induced sympathetic blockade, fluid loading, repeated phenylephrine boluses, and oxytocin - supports this interpretation: neuraxial sympathectomy markedly alters autonomic balance, and phenylephrine-induced baroreflex bradycardia can slow atrioventricular nodal conduction, thereby favoring preferential conduction along the accessory pathway. The normal antepartum ECG, recorded under stable conditions, is fully consistent with this hypothesis.

The second hypothesis is a cardiovascular manifestation of mast cell activation. Although a spectrum of cardiovascular manifestations of mast cell mediator release has been described [4,19], no established evidence links mast cell disease to the de novo appearance of an accessory-pathway pre-excitation pattern, and any such association would at present be purely speculative. Moreover, the consistently normal perioperative tryptase values and the absence of any clinical signs of mast cell activation make this mechanism highly unlikely in the present case.

The third hypothesis is the Kounis syndrome (allergic acute coronary syndrome) [20]. This was considered and excluded: the patient had no chest pain, troponin and echocardiography were unremarkable, and tryptase remained normal; the pre-excitation pattern was also mechanistically distinct from the ischemic ST-T changes characteristic of Kounis syndrome.

The pragmatic implications are twofold. First, the case underscores the importance of a preoperative baseline ECG and of vigilant intraoperative electrocardiographic monitoring in this population, both for early detection of mast cell-related cardiovascular events and for the recognition of newly apparent arrhythmogenic substrates. Second, it expands the range of cardiovascular phenomena that may be observed in the perioperative care of patients with SM and reinforces the importance of close collaboration with cardiology when unexpected findings emerge. The recognition of a pre-excitation pattern is clinically actionable, allowing outpatient electrophysiological risk stratification and informing the safety of future anesthetic procedures.

Limitations

Several limitations should be acknowledged. First, this is a single case report, and although it provides objectively documented outcomes, its findings cannot be generalized and do not carry the evidentiary weight of prospective data. The perioperative strategy adopted was necessarily based on the best available evidence, which in this field is largely limited to case reports, retrospective series, and expert consensus, in the absence of large-scale prospective studies in this specific patient population. Second, regarding the intraoperative pre-excitation pattern, no acute electrophysiological study was performed to confirm the presence and characteristics of an accessory pathway; the proposed mechanism, therefore, remains a plausible but unconfirmed hypothesis-generating interpretation rather than a definitively established finding. An outpatient electrophysiological evaluation was arranged to address this point. Finally, the favorable biochemical and clinical course observed reflects a single patient with indolent SM and may not be reproducible across the broader spectrum of mast cell disorders or in emergency settings.

## Conclusions

This report describes the successful perioperative management of a patient with SM and latex allergy undergoing elective cesarean section, complicated by the unexpected intraoperative appearance of a WPW pre-excitation pattern. The key lessons are the centrality of multidisciplinary collaboration, the primacy of a prevention-oriented anesthetic strategy, and the importance of pharmacologically informed drug selection at every stage of the perioperative pathway. Our report supports spinal anesthesia with hyperbaric 0.5% bupivacaine (12 mg) as a reasonable preferred option for this indication when not contraindicated, combining effective surgical anesthesia with a favorable mast cell safety profile. Intraoperative hypotension was safely managed with phenylephrine boluses, whose selective alpha-1 mechanism is preferable to indirect sympathomimetics in this context. The normality of perioperative serum tryptase supported the effectiveness and safety of the preventive strategy. Postoperative analgesia with paracetamol, excluding NSAIDs, proved clinically adequate and pharmacologically appropriate. The asymptomatic intraoperative emergence of a new pre-excitation pattern - most plausibly attributable to the unmasking of a pre-existing concealed accessory pathway by perioperative autonomic and pharmacological shifts - broadens the spectrum of intraoperative electrocardiographic findings to be considered in this population and reinforces the importance of vigilant intraoperative cardiac monitoring and prompt cardiology consultation.
